# Path Planning for Dragon-Fruit-Harvesting Robotic Arm Based on XN-RRT* Algorithm

**DOI:** 10.3390/s25092773

**Published:** 2025-04-27

**Authors:** Chenzhe Fang, Jinpeng Wang, Fei Yuan, Sunan Chen, Hongping Zhou

**Affiliations:** College of Mechanical and Electronic Engineering, Nanjing Forestry University, Nanjing 210037, China; fangcz@njfu.edu.cn (C.F.);

**Keywords:** pitaya harvesting, picking robotic arm, path planning, normal distribution, XN-RRT* algorithm

## Abstract

This paper proposes an enhanced RRT* algorithm (XN-RRT*) to address the challenges of low path planning efficiency and suboptimal picking success rates in complex pitaya harvesting environments. The algorithm generates sampling points based on normal distribution and dynamically adjusts the center and range of the sampling distribution according to the target distance and tree density, thus reducing redundant sampling. An improved artificial potential field method is employed during tree expansion, incorporating adjustment factors and target points to refine the guidance of sampling points and overcome local optima and infeasible targets. A greedy algorithm is then used to remove redundant nodes, shorten the path, and apply cubic B-spline curves to smooth the path, improving the stability and continuity of the robotic arm. Simulations in both two-dimensional and three-dimensional environments demonstrate that the XN-RRT* algorithm performs effectively, with fewer iterations, high convergence efficiency, and superior path quality. The simulation of a six-degree-of-freedom robotic arm in a pitaya orchard environment using the ROS2 platform shows that the XN-RRT* algorithm achieves a 98% picking path planning success rate, outperforming the RRT* algorithm by 90.32%, with a 27.12% reduction in path length and a 14% increase in planning success rate. The experimental results confirm that the proposed algorithm exhibits excellent overall performance in complex harvesting environments, offering a valuable reference for robotic arm path planning.

## 1. Introduction

With the deployment of fruit-picking robots in more complex environments, the optimization of automation and intelligence within these processes has become critical for enhancing production efficiency and reducing labor costs [1]. The harvesting of a diverse range of fruits, from apples and oranges to dragon fruit, can no longer meet the increasing demands placed on traditional manual operations [2]. Consequently, studying the path planning algorithms for the picking manipulator in complex fruit growth environments is of considerable importance [3,4,5,6].

To mitigate the influence of branches and other obstacles on the robotic arm’s trajectory during the picking process, research on obstacle avoidance path planning algorithms for the robotic arm is crucial. Commonly employed obstacle avoidance path planning algorithms include the Dijkstra algorithm, A* algorithm, artificial potential field method (APF), genetic algorithms, and the RRT algorithm [7,8,9,10]. While the Dijkstra algorithm guarantees an optimal solution, it suffers from high computational complexity and low efficiency in large-scale picking environments [11]. The A* algorithm, when combined with the Dijkstra algorithm and heuristic search, can enhance computational efficiency; however, its reliance on heuristic functions may hinder search performance in certain complex picking environments [12]. The artificial potential field method exhibits strong obstacle avoidance capabilities, but is prone to falling into local minima [13]. Genetic algorithms, while effective for complex problems, tend to converge slowly [14]. In contrast, the RRT algorithm efficiently identifies paths in high-dimensional spaces through random sampling and tree expansion, making it widely used in obstacle avoidance planning for picking manipulators. However, it relies heavily on random sampling and lacks a global planning mechanism during node expansion, resulting in low path planning efficiency and excessive node generation, which negatively impacts overall performance [15,16,17]. As a result, the algorithm exhibits certain limitations in practical applications. To address these limitations, numerous researchers have proposed various improvement strategies aimed at enhancing its performance.

The RRT algorithm lacks global optimality and may fail to identify the path with the minimum cost. In response, KARAMAN et al. [18] introduced an optimization mechanism, resulting in the RRT* algorithm, which dynamically reselects parent nodes and continuously optimizes paths during tree expansion, gradually achieving the global optimal path. However, the RRT* algorithm, due to its random sampling strategy and lack of effective local search and guidance mechanisms, suffers from a low sampling efficiency in high-dimensional picking spaces, slow path planning speed, and path jitter. In the domain of picking-robot path planning, Song Yong et al. [19] proposed the PAPF-RRT* algorithm, which optimizes sampling positions by introducing target bias probability and optimal random sampling point selection strategies, thereby enhancing guidance. Li Na et al. [20] proposed an improved integrated switching path dynamic programming algorithm, which sets thresholds to segment the picking area and dynamically switches to the FGA-RRT algorithm in narrow passage areas, improving both convergence speed and planning success rate. Xiong Juntao et al. [21] addressed the disorder of the lychee growth environment by proposing the PIB-RRT* algorithm, which uses the artificial potential field method for spatial segmentation and four-way search, combines potential field guidance with random sampling, improves sampling quality, and realizes adaptive dynamic step expansion based on node historical expansion information. Li et al. [22] proposed the AS-RRT* algorithm, which adopts a sampling point selection strategy based on accumulators to improve path planning efficiency and ensures real-time obstacle avoidance for the picking robot by a dynamic step length adjustment mechanism after collision detection. Zhang Qin et al. [23] proposed the CTB-RRT* algorithm, which optimizes sampling blindness, local search speed, and computational efficiency through Cauchy distribution heuristic sampling, the dynamic adjustment of step length, and a node rejection strategy.

In summary, to address the issues of high sampling randomness, low planning efficiency, and path jitter inherent in the traditional RRT* algorithm, this paper proposes the XN-RRT* algorithm, building upon improvements to the RRT* algorithm. Sampling points are generated using a normal distribution, with both the sampling center and range dynamically adjusted, thereby mitigating the issue of repetition in random sampling. During tree expansion, combined with the improved artificial potential field method for guided sampling, the sampling orientation is enhanced by incorporating moderating factors and target points, effectively addressing local optimality and reachability issues. Finally, a greedy algorithm is employed to eliminate redundant nodes, shorten path length, and smooth the trajectory using cubic B-spline curves, thereby enhancing both the smoothness and continuity of robotic arm movement.

## 2. Robotic Arm Path Planning and Collision Detection

### 2.1. Path Planning Definition

In the context of path planning, consider a space that encompasses all potential states. The region occupied by obstacles is referred to as the obstacle space, which is excluded from the overall space, thereby yielding a region devoid of obstacles, commonly known as a free space or obstacle-free space. Within this obstacle-free space, given an initial position and a target position, the objective of path planning is to identify a continuous, collision-free path that connects the start and target positions, while satisfying the necessary conditions for traversal [24]. The objective of the optimal path planning problem is to identify the path that minimizes the cost among all collision-free paths that satisfy the required planning conditions [25].

### 2.2. Collision Detection Description

Collision detection plays a pivotal role in robotic arm path planning, serving as a fundamental step to ensure the safety of the motion trajectories. The primary objective of collision detection is to determine whether the robotic arm’s motion trajectory intersects with any obstacles or its own structure, thereby enabling the path planning algorithm to generate collision-free trajectories [26].

**Figure 1:**
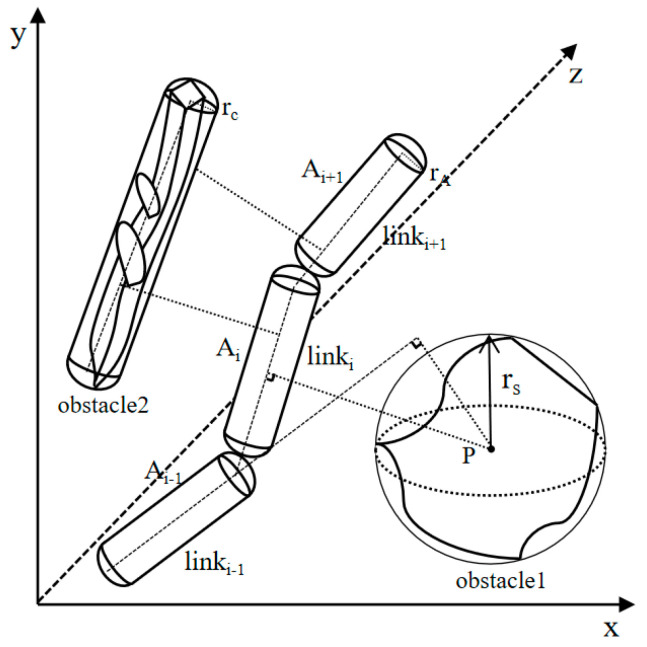
Simplified collision detection model.

Given that obstacles and robotic links are often irregular geometric entities in practical applications, this study adopts the geometric envelope method to facilitate their modeling [27]. Specifically, irregular obstacles are represented by spheres or spherical cylinders, whereas robotic links are characterized as spherical cylinders. The simplified collision detection model is illustrated in Figure 1.

Following the aforementioned simplification of the collision model, the problem of collision detection between the robotic arm and the obstacle can be reduced to determining the geometric relationship between the spherical cylinder and the sphere, as well as the spherical cylinder.

In order to streamline the computational process, we assume that the radius of the robotic arm link, modeled as a spherical cylinder, is denoted as $r_{A}$, with the axis represented by the line segment $A_{1}A_{2}$. The radius of the obstacle, modeled as a sphere, is denoted as $r_{S}$, with the center located at point P. Additionally, the radius of the obstacle, modeled as a spherical cylinder, is denoted as $r_{C}$, with its axis represented by the line segment $C_{1}C_{2}$. Building upon the methodology outlined in [28], the constraints are formulated as a system of nonlinear inequalities, and the collision conditions are subsequently translated into distance-based judgments within the geometric space.

Collision detection between the robotic arm and a spherical obstacle is a matter of identifying events that can be classified into two distinct cases: (1) when the projection of the sphere’s center lies within the actual boundaries of the spherical cylinder; (2) when the projection of the sphere’s center falls along the axis extension direction of the spherical cylinder. To simplify collision detection, the projection of the sphere’s center is parameterized as follows:

(1) $$t=\frac{\left(P-A_{1}\right)\cdot \left(A_{2}-A_{1}\right)}{{\left\|A_{2}-A_{1}\right\|}^{2}}$$

The conditions for the robot arm to collide with a spherical obstacle are as follows:

(2) $$d_{min}=\left\{\begin{array}{c} \frac{\left\|\left(P-A_{1}\right)\times \left(A_{2}-A_{1}\right)\right\|}{\left\|A_{2}-A_{1}\right\|}\;\;\;\;\;\;\;\;\;\;\;\;\;\;\;\;\;\;\;\;\;\;\;t\in \left[0,1\right]\\ \;\;\;\;\;\;\;\left\|P-A_{1}\right\|\;\;\;\;\;\;\;\;\;\;\;\;\;\;\;\;\;\;\;\;\;\;\;\;\;\;\;\;\;\;\;\;\;\;\;\;\;t<1\\ \;\;\;\;\;\;\;\left\|P-A_{2}\right\|\;\;\;\;\;\;\;\;\;\;\;\;\;\;\;\;\;\;\;\;\;\;\;\;\;\;\;\;\;\;\;\;\;\;\;\;\;t>1 \end{array}\right.\leq r_{A}+r_{S}$$

Collision assessment between the robotic arm and a spherical–cylindrical obstacle can be categorized into two scenarios: (1) when the axis of the spherical–cylindrical obstacle aligns with the axis of the robotic arm’s link; (2) when the axis of the spherical–cylindrical obstacle does not align with the axis of the robotic arm’s link.

In a case where the axis of the spherical–cylindrical obstacle is aligned with the axis of the robotic arm, the coordinate system should be adjusted to rotate until the two axes become perpendicular to a plane. Under these conditions, the collision criterion is as follows:

(3) $$D_{min}=\sqrt{u_{1}^{2}+u_{2}^{2}}\leq r_{A}+r_{C}$$

where $D_{min}$ is the shortest distance between the robot arm and the axis of the spherical–cylindrical obstacle, $u_{1}$ is the distance within the plane between the axis of the projected robotic arm and the axis of the spherical–cylindrical obstacle, and $u_{2}$ is the axial spacing between the axis of the robotic arm and the axis of the spherical–cylindrical obstacle.

If the axis of the spherical–cylindrical obstacle is not aligned with the axis of the robotic arm’s connecting rod, an auxiliary plane is constructed based on the axis of the obstacle ($C_{1}C_{2}$) to align it with axis $A_{1}A_{2}$. The resulting collision conditions are as follows:

(4) $$D_{min}=\sqrt{u_{3}^{2}+u_{4}^{2}}\leq r_{A}+r_{C}$$

where $u_{3}$ is the distance between axis $A_{1}A_{2}$ and the auxiliary plane and $u_{4}$ is the minimum distance between the projection of axis $A_{1}A_{2}$ onto the auxiliary plane and axis $C_{1}C_{2}$.

## 3. XN-RRT* Algorithm

To address the challenges encountered in the intricate picking environment of the RRT* algorithm, this study introduces the XN-RRT* algorithm, which enhances the three critical stages of sampling space, new node expansion, and path optimization. It reduces the search time and path length, effectively diminishes the number of required sampling points, optimizes node utilization, and produces smoother motion trajectories.

### 3.1. Based on Normal Distribution Dynamic Sampling Method

In contrast to the RRT* algorithm, which utilizes random sampling across a global workspace, this paper introduces a dynamic sampling approach grounded in normal distribution principles. This method leverages the inherent properties of the normal distribution to optimize the sampling space within the RRT* algorithm. In the context of path planning, the normal distribution is employed to generate sampling points, with these points adhering to a normal distribution. The expression for this distribution is as follows:

(5) X=μ+σ⋅N(0,1)

where μ is the mean (sampling distribution center) and σ is the standard deviation (sampling range).

In the expansion Formula (3) of the tree, the standard normal distribution Z∼N(0,1) represents the normalized noise of the normal distribution. This signifies that the tree’s expansion is primarily focused around the mean. As the standard deviation decreases, the distribution of sampling points becomes progressively more concentrated. In the context of path planning, utilizing this standard normal distribution as the fundamental noise source allows for the flexible generation of sampling points X∼N(μ,σ) that align with the target distribution, depending on the current position of the tree node and the target point.

By dynamically adjusting the sampling distribution center and incorporating a mixed strategy that is also dynamically adjusted within the sampling range, the efficiency of sampling and the speed of path convergence can be further enhanced.

During the initial phase of node expansion, the sampling distribution center is set at the most recent node of the tree $X_{new}$. A larger initial standard deviation is then employed to facilitate expansion along the tree’s boundary, explore new path branches, cover a broader workspace, and avoid local optima. During the target point guidance phase, the sampling distribution center is adjusted to the target point after every fixed number k of iterations (this study sets k to 10). The standard deviation is subsequently reduced, concentrating sampling points around the target $X_{goal}$. This adjustment enhances the exploration capability of the target area and accelerates the convergence of the tree. In subsequent iterations, at fixed intervals, the directional vector from the latest node of the current tree to the target point is calculated, normalized to a unit vector, and used to generate a new sampling point based on the direction and step length. This sampling point is then established as the new sampling distribution center μ, ensuring that the μ adjustment is dynamic and gradually approaches the target point. The mathematical expression for the sampling distribution center is as follows:

(6) $$\mu =\left\{\begin{array}{c} X_{goal},\;\mathrm{if}\;i\;\mathrm{mod}\;k=0\\ X_{new},\;\mathrm{otherwise}\;\;\;\;\;\;\;\;\; \end{array}\right.$$

where i is the current iteration count and  k is the interval adjustment frequency.

The dynamic adjustment of the mixed strategy, grounded in the standard deviation, facilitates a flexible modulation of the sampling range, which is tailored to the target distance and tree density, thereby enhancing the efficiency of path planning. The expression for the sampling range σ is as follows:

(7) $$\sigma =\left\{\begin{array}{c} \sigma_{min},\;\mathrm{if}\;\mu =X_{goal}\;\;\;\;\\ \max(\sigma_{min},\;min(\sigma_{dis},\sigma_{den})),\;if\;\mu =X_{new} \end{array}\right.$$

where $\sigma_{min}$ is the dynamic adjustment of the minimum standard deviation limit, $\sigma_{dis}$ is the dynamic adjustment of standard deviation with distance factor, and $\sigma_{den}$ is the dynamic adjustment of the standard deviation with a tree density factor.

The $\sigma_{dis}$ decreases as the path approaches the target, initially being larger to facilitate extensive exploration and gradually decreasing to enhance path accuracy. This can be expressed as follows:

(8) $$\sigma_{dis}=\sigma_{max}\cdot \frac{\rho (X_{near},X_{goal})}{\rho (X_{init},X_{goal})}$$

where $\sigma_{max}$ is the dynamic adjustment of the maximum standard deviation limit, $X_{goal}$ is the target point position, $X_{near}$ is the expanded tree to the nearest node position, $\rho (X_{near},X_{goal})$ is the Euclidean distance between the recent node and the target point, and $\rho (X_{init},X_{goal})$ is the Euclidean distance between the initial node and the target point.

As the tree density increases, the standard deviation should decrease to prevent over-sampling. Tree density can be computed based on the number of nodes and the spatial area size. The $\sigma_{den}$ can be expressed as follows:

(9) $$\sigma_{den}=\sigma_{max}\cdot (1-\frac{P(X_{near})}{P_{max}})$$

where $P(X_{near})$ is the current node density within the region and $P_{max}$ is the maximum node density reached by the trees in the densest area.

**Figure 2:**
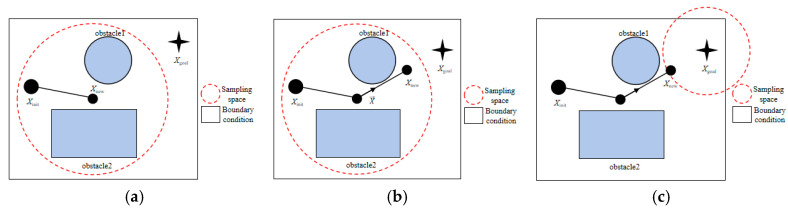
Sampling schematic diagram. (**a**) Initial sampling period; (**b**) goal-oriented period 1; (**c**) goal-oriented period 2.

The sampling schematic diagram is shown in Figure 2.

By employing this dynamic adjustment strategy for both the sampling center and range, the XN-RRT* algorithm is able to extensively explore the entire search space during the initial phase, thereby mitigating the risk of converging to local optima. In the later stages, it intensifies local exploration, rapidly approaching the target and thereby accelerating the identification of a feasible path from the initial node to the target point.

### 3.2. Improved Artificial Potential Field Method-Assisted Guided Sampling Strategy

A dynamic sampling approach utilizing normal distribution can significantly mitigate the occurrence of invalid samples. However, in certain complex environments, the path may converge to a local minimum, resulting in diminished efficiency or suboptimal path quality. To enhance the efficiency of the sampling process in the XN-RRT* algorithm, this study presents an auxiliary guidance sampling strategy integrated with the enhanced artificial potential field method.

The artificial potential field method is a path planning approach grounded in the principles of physics and mechanics. The core concept involves treating the target point and obstacles as attractive and repulsive sources, respectively, and determining the motion direction of the robotic arm by calculating the resultant forces exerted on it, thereby optimizing the path planning process. In path planning algorithms that rely on random sampling, the traditional artificial potential field method is frequently employed for guidance. However, when employing the traditional artificial potential field method for guiding sampling, the robotic arm may encounter a scenario in which the attractive force diminishes to zero while the repulsive force persists when the target point is reached, resulting in the problem of an unreachable target. Moreover, if the resultant direction of all obstacle repulsive forces aligns with that of the attractive force, and the robotic arm has not yet reached the target point, the system may encounter a condition where the repulsive and attractive forces are equal, thus leading to local optimization.

To enhance the traditional artificial potential field method, a regulating factor $k_{g}$ is introduced into the repulsive field model of obstacles, ensuring that the repulsive and attractive forces diminish to zero concurrently only when the robotic arm reaches the target point. This modification effectively addresses the issues of local optimality and unreachable target points. The mathematical formulation of the regulating factor $k_{g}$ is presented as follows:

(10) $$k_{g}=1-e^{\frac{-\rho^{\alpha }(X_{near},X_{goal})}{W_{max}}}$$

where α is the adjustment parameters (constants) and $W_{max}$ is the working space radius of the robotic arm.

Leveraging the enhanced artificial potential field method, the robotic arm navigates toward the target point under the simultaneous influence of both the attractive potential field and the modified repulsive potential field. During this process, owing to the concurrent influence of the repulsive fields from multiple obstacles, the repulsive force acting on the robotic arm is the vector sum of the repulsive forces from each individual obstacle. Consequently, the resultant potential field encountered by the robotic arm can be mathematically expressed as follows:

(11) $$U_{total}=U_{att}+\sum_{i=0}^{n}U_{rep\left(i\right)}$$

where $U_{att}$ is the attractive potential field function and $U_{rep}$ is the repulsive potential field function.

The expression of the resultant force is as follows:

(12) $$F_{total}=F_{att}+\sum_{i=0}^{n}F_{rep\left(i\right)}$$

The repulsive potential field function of the improved artificial potential field method is expressed as:

(13) $$U_{rep}=\left\{\begin{array}{c} \frac{1}{2}k_{rep}{k_{g}(\frac{1}{\rho (X_{near},X_{obs})}-\frac{1}{\rho_{0}})}^{2},0\leq \rho (X_{near},X_{obs})\leq \rho_{0}\\ 0,\rho (X_{near},X_{obs})\geq \rho_{0} \end{array}\right.$$

where $k_{rep}$ is the repulsion gain coefficient, $X_{obs}$ is the obstacle position, $\rho (X_{near},X_{obs})$ is the Euclidean distance between the recent node position and the obstacle position, and $\rho_{0}$ is the range (constant) of the repulsive potential field.

In the calculation of the repulsive force exerted by obstacles, this study further incorporates the influence of the target point, thereby adjusting the direction of the repulsive force not only to point towards the obstacle but also to be modulated by the attraction of the target point. This modification ensures that the robotic arm remains on course towards the target, even when encountering a strong repulsive force from obstacles during the avoidance process. This dynamically adjusted repulsive force field enhances the flexibility of path planning, enabling it to adapt to the variability of complex picking environments.

(14a) $$F_{rep}=-\nabla U_{rep}=\left\{\begin{array}{c} F_{rep1}+F_{rep2},0\leq \rho (X_{near},X_{obs})\leq \rho_{0}\\ 0,\rho (X_{near},X_{obs})\geq \rho_{0} \end{array}\right.$$

(14b) $$\left\{\begin{array}{c} F_{rep1}=k_{rep}k_{g}(\frac{1}{\rho (X_{near},X_{obs})}-\frac{1}{\rho_{0}})\frac{\nabla \rho (X_{near},X_{obs})}{\rho^{2}(X_{near},X_{obs})}\\ F_{rep2}=\frac{1}{2}k_{rep}{\left(\frac{1}{\rho (X_{near},X_{obs})}-\frac{1}{\rho_{0}}\right)}^{2}\nabla k_{g} \end{array}\right.$$

The direction of $F_{rep1}$ is from $X_{obs}$ to $X_{near}$ and the direction of $F_{rep2}$ is from $X_{near}$ to $X_{goal}$.

The attractive potential function $U_{att}$ in the improved artificial potential field method is the same as that in the traditional artificial potential field method, and its expression is as follows:

(15) $$U_{att}=\frac{1}{2}k_{att}\rho^{2}(X_{near},X_{goal})$$

where $k_{att}$ is the attraction gain coefficient.

The attractive force $F_{att}$ is the negative gradient of the attractive force potential field, and its expression is as follows:

(16) $$F_{att}=-\nabla U_{att}=k_{att}\rho \left(X_{near},X_{goal}\right)$$

**Figure 3:**
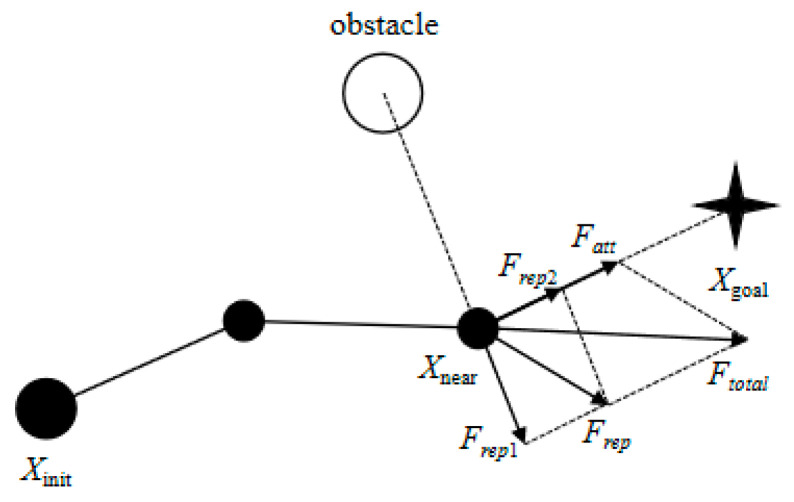
An improved artificial potential field guidance sampling diagram.

An improved artificial potential field guidance sampling diagram is shown in Figure 3.

By analyzing Equations (10), (14a), and (14b), along with the enhanced artificial potential field guidance sampling diagram, it is evident that the relationship between the current node and the target node is incorporated into the repulsive potential field function, while the influence of the target point is integrated into the calculation of the obstacle’s repulsive force. This results in the direction of the repulsive force being influenced not only by the obstacle but also adjusted by the attractive force of the target point. Consequently, when the robotic arm reaches the target point, both the attractive and repulsive forces synchronize to zero, thereby effectively mitigating issues related to local optima and the inaccessibility of targets.

### 3.3. Path Optimization Method

The path generated through the enhanced path planning approach exhibits numerous sharp turns, resulting in a winding and uneven trajectory. To optimize the quality of the generated path, this study introduces a greedy-algorithm-based strategy to eliminate redundant nodes, thereby minimizing the number of linear segments in the trajectory, which simplifies the overall path structure and enhances both the stability and efficiency of robotic arm movement. The core principle underlying this approach is as follows:

Add the initial point $X_{init}$ of the path to the new path ${Path}_{new}$ and set it as the current detection point $X_{cur}$:

(17) $$\left\{\begin{array}{c} {Path}_{new}=\left\{X_{init}\right\}\\ X_{cur}=X_{init} \end{array}\right.$$

Check whether the connection between the current detection point $X_{cur}$ and the next node $X_{next}$ in the original path ${Path}_{old}$ collides with obstacles; if it does not, continue to check the connections between subsequent nodes $X_{cur}$ to ${Path}_{old}$.

When a collision is detected between the connection from the current detection point $X_{cur}$ to node $X_{stop}$, node $X_{last}$ on the previous path of $X_{stop}$ is added to the new path and set as the new detection point:

(18) $$\left\{\begin{array}{c} {Path}_{new}=\left\{X_{init},X_{last}\right\}\\ X_{cur}=X_{last} \end{array}\right.$$

Repeat steps (2) and (3) until detection point $X_{cur}$ becomes target point $X_{goal}$, and add $X_{goal}$ to the new path ${Path}_{new}$:

(19) $${Path}_{new}=\left\{X_{init},\cdots ,X_{goal}\right\}$$

**Figure 4:**
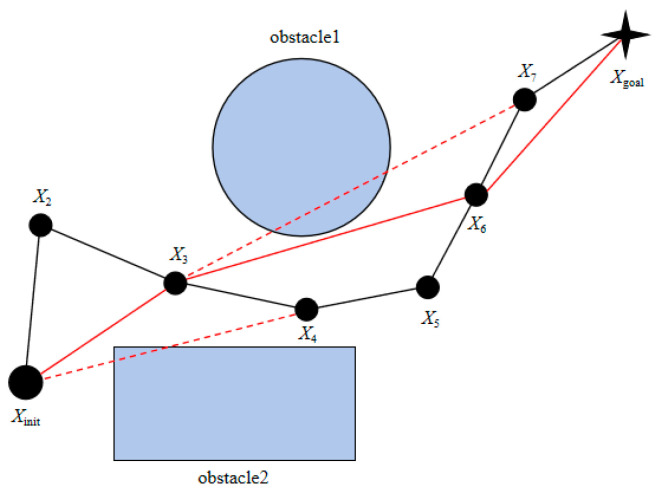
Greedy algorithm to remove redundant nodes.

As shown in Figure 4, the black line is the original path, the red dashed line is the collision detection, and the red line is the new optimized path. Starting from the starting point $X_{init}$ of the path, check the node set in the path sequentially. The connection between $X_{init}$ and node $X_{2}$ and the connection between $X_{init}$ and node $X_{3}$ do not collide with obstacles. A collision occurs between $X_{init}$ and node $X_{4}$, so add node $X_{3}$ to the new path; then, take node $X_{3}$ as the detection point, and find that the connections between node $X_{3}$ and node $X_{4}$, node $X_{3}$ and node $X_{5}$, and node $X_{3}$ and node $X_{6}$ do not collide with obstacles. A collision occurs between node $X_{3}$ and node $X_{7}$, so add node $X_{6}$ to the new path; then, take node $X_{6}$ as the detection point, find that the connections between node $X_{6}$ and node $X_{7}$ and node $X_{6}$ and node $X_{goal}$ do not collide with obstacles, and node add $X_{goal}$ to the new path. Therefore, the optimized new path is as follows:

(20) $${Path}_{new}=\left\{X_{init},\;X_{3},\;X_{6},\;X_{goal}\right\}$$

After eliminating redundant points from the path, the overall path quality has been improved; however, a few turning points remain in the optimized trajectory. To ensure both the smoothness and effectiveness of the robotic arm’s path, further smoothing is required. Consequently, cubic B-spline curves are employed in this study to smooth the optimized trajectory, with the following mathematical formulation:

(21) $$C\left(u\right)=\sum_{i=0}^{n}N_{i,3(u)}\cdot P_{i},u\epsilon [u_{k},u_{k+1}]$$

where $C\left(u\right)$ is the point on the curve under the curve parameter u and the curve is composed of multiple segments, $P_{i}$ is the control points of a curve—usually the target points and inflection points of the path, and $N_{i,3(u)}$ is the cubic B-spline basis function.

**Figure 5:**
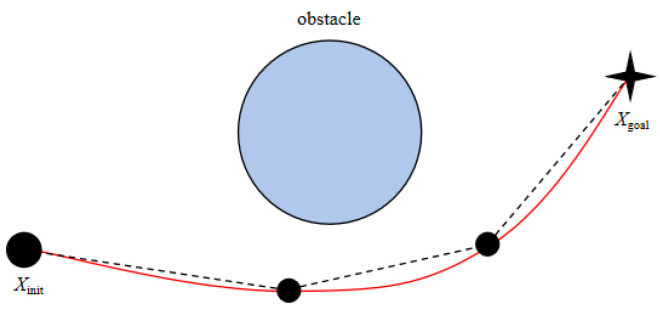
Cubic B-spline curve optimization path schematic.

The smoothed curve is shown in Figure 5.

**Figure 6:**
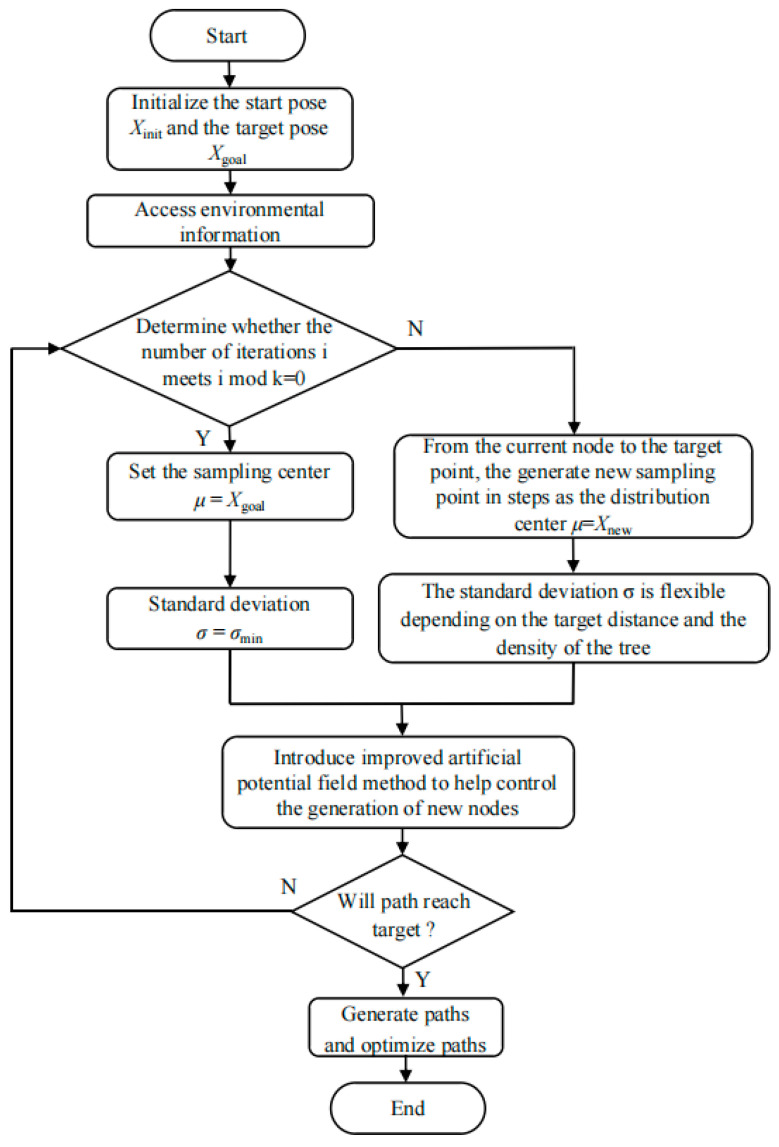
XN-RRT* algorithm flow chart.

The overall process of the algorithm is shown in Figure 6.

## 4. Algorithm Simulation and Analysis

To assess the effectiveness of the proposed XN-RRT* algorithm, comparative simulation experiments and analyses were performed on the traditional RRT* algorithm, the RRT* variant enhanced by integrating the conventional artificial potential field method (APF-RRT* algorithm [29]), and the XN-RRT* algorithm under a variety of distinct scenarios. The experimental setup for the comparative simulations was configured with an NVIDIA GeForce RTX 3050 Laptop GPU, an AMD Ryzen 5 6600H processor, 16 GB of memory, and a 64-bit Windows 11 operating system.

### 4.1. Two-Dimensional Space Simulation and Analysis

**Figure 7:**
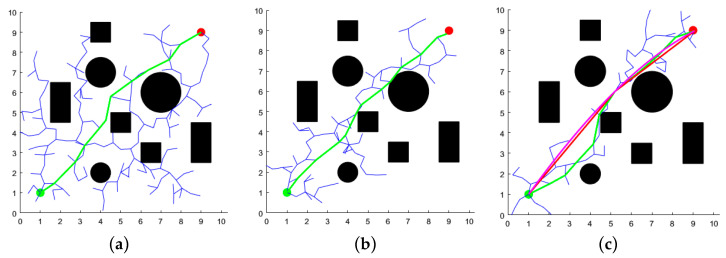
Results of three algorithms for path planning in simple discrete environment. (**a**) RRT* algorithm; (**b**) APF-RRT* algorithm; (**c**) XN-RRT* algorithm.

**Figure 8:**
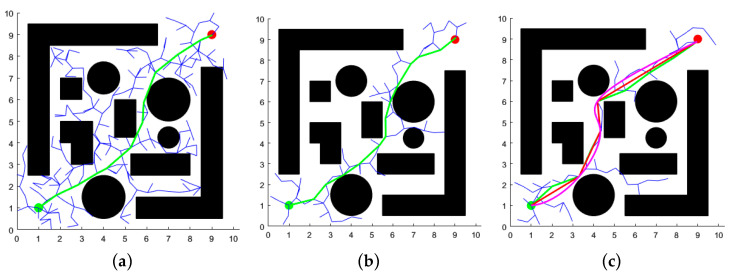
Results of three algorithms for path planning in complex narrow environment. (**a**) RRT* algorithm; (**b**) APF-RRT* algorithm; (**c**) XN-RRT* algorithm.

In MATLABR2022b, two distinct two-dimensional map environments were constructed: one representing a simple discrete layout and the other a complex narrow configuration. The map size was set to 10 m × 10 m, with the starting point located at (1, 1) m and the target point at (9, 9) m. The maximum number of iterations for the three algorithms was set to 2000, with a search step size of 0.5 m and a distance threshold of 0.3 m for the target point. The experimental results are presented in Figure 7 and Figure 8, where the black circles and rectangles represent obstacles, green curves denote the initial paths, red indicates redundant path removal, purple corresponds to the final path, blue illustrates the branches of the random tree, green dots mark the starting points, and red dots denote the target points of the path.

As evidenced by Figure 7 and Figure 8, the XN-RRT* algorithm exhibits more pronounced directional characteristics during the path search process, producing paths that are not only more concise and smoother, but also exhibit reduced redundancy. Furthermore, the algorithm demonstrates enhanced exploration capabilities in confined spaces. Given the variability in path planning across iterations, each algorithm was executed 100 times within the two environments. The resulting average values of path length, runtime, and iteration count are presented in Table 1.

The XN-RRT* algorithm demonstrates comparable path length performance when compared to the traditional RRT and APF-RRT* algorithms; however, it exhibits substantial improvements in terms of both computational time and iteration count. In simple, discrete environments, the XN-RRT* algorithm achieves a reduction in running time by approximately 83.5% and 47.6% compared to the RRT* and APF-RRT* algorithms, respectively, while iteration counts are reduced by approximately 80.8% and 60.3%. In complex, constrained environments, the XN-RRT* algorithm reduces running time by approximately 85.0% and 55.3% relative to the RRT and APF-RRT* algorithms, respectively, while iteration counts are reduced by approximately 78.9% and 54.7%. This further substantiates the efficacy of the enhanced algorithm.

In conclusion, the XN-RRT* algorithm not only enhances planning efficiency but also optimizes the overall performance of path planning, all while maintaining comparable path quality to traditional algorithms.

### 4.2. Three-Dimensional Space Simulation and Analysis

**Figure 9:**
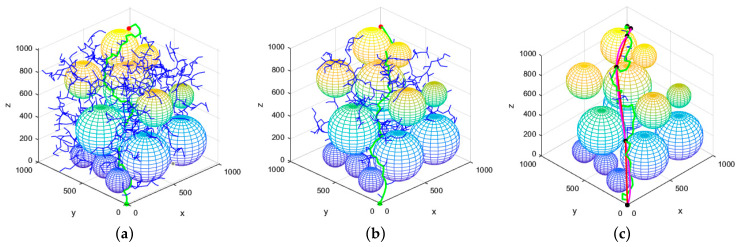
Results of three algorithms for path planning in 3D environment. (**a**) RRT* algorithm; (**b**) APF-RRT* algorithm; (**c**) XN-RRT* algorithm.

In order to evaluate the effectiveness of the proposed algorithm in three-dimensional (3D) environments, a simulation map was constructed using MATLAB. The map’s dimensions were set to 1000 m × 1000 m × 1000 m, with the initial point located at (0, 0, 0) m and the target point at (900, 900, 900) m. The maximum number of iterations for the three algorithms was set to 5000, with a search step size of 50 m and a target point distance threshold of 30 m. The experimental results are presented in Figure 9.

As depicted in Figure 9, the conventional RRT* algorithm produces a substantial quantity of redundant, invalid nodes, primarily attributable to the absence of a well-defined goal-directedness in the random tree expansion process. Consequently, the resultant path exhibits an increased number of sharp turns, resulting in suboptimal overall smoothness. The APF-RRT* algorithm enhances the directionality of random tree expansion by incorporating an artificial potential field model, thereby mitigating the occurrence of invalid nodes to a certain degree. Nevertheless, the path retains a relatively pronounced winding structure. In contrast, the XN-RRT* algorithm demonstrates a more pronounced goal-directedness during random tree expansion, resulting in a significant reduction in the generation of invalid nodes and further optimizing the path shape, thereby leading to a notable decrease in the number of sharp turns. The resulting path exhibits enhanced smoothness and superior quality.

In three-dimensional space, 100 repeated simulation experiments were performed for each algorithm. The corresponding average values for the generated path length, computation time, and number of iterations are presented in Table 2.

Table 2 presents the results, indicating that, within a three-dimensional environment, the enhanced RRT* algorithm reduces path length by approximately 14.99% relative to the original RRT* algorithm and by about 6.23% in comparison to the APF-RRT* algorithm. Additionally, the computational runtime is reduced by approximately 96.21% and 88.55%, respectively, while the number of iterations decreases by around 93.29% and 86.52%, respectively.

In conclusion, the efficacy of the enhanced RRT* algorithm in three-dimensional environments has been further substantiated. Through augmenting the goal-directed expansion of the random tree, the algorithm significantly mitigates the formation of invalid nodes while expediting its convergence rate. Moreover, the trajectories generated through the path optimization method exhibit superior quality and enhanced smoothness, thereby highlighting its advantages in tackling complex spatial planning challenges.

## 5. Simulation Environment Picking Test and Analysis

**Figure 10:**
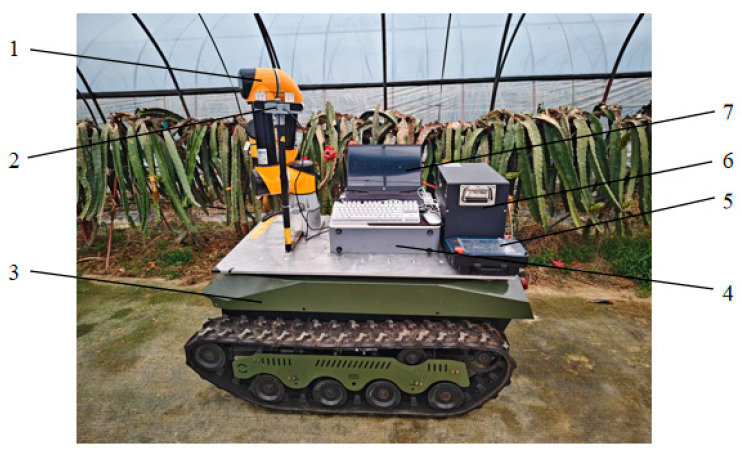
Dragon-fruit-picking robot. (1: robotic arm; 2: ZED binocular camera; 3: tracked chassis car; 4: control cabinet; 5: teaching pendant; 6: power supply; 7: host computer).

In order to assess the efficacy of the XN-RRT* algorithm in the context of dragon fruit harvesting, and to validate its feasibility, this study developed a prototype of a robotic system designed for dragon fruit picking, as illustrated in Figure 10. Within the ROS2 framework, the robot arm parameters and initialization files were configured using Moveit2, while the algorithm presented in this study was integrated into the Open Motion Planning Library (OMPL). Furthermore, the dragon fruit harvesting scenario was modeled using SolidWorks2021 and subsequently imported into Rviz for collision detection, enabling obstacle avoidance path planning within the simulated environment.

**Figure 11:**
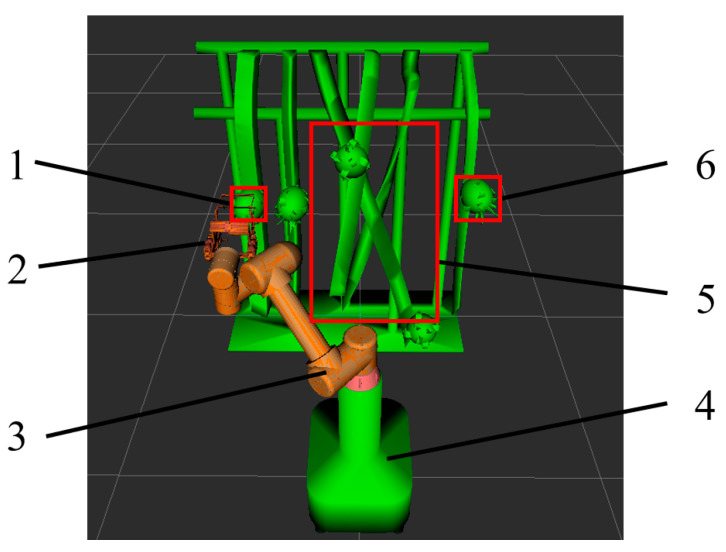
Simulation of dragon fruit picking environment. (1: initial position of robotic arm; 2: picking end-effector; 3: robotic arm; 4: Base; 5: branches and fruit obstacles; 6: target fruit).

The obstacle model within the simulation environment was simplified, as shown in Figure 11, with the robot arm’s initial joint angles configured as (0.873, −0.785, 1.571, 2.356, −2.443, 2.618) radians while the target joint angles were set to (−1.222, −0.873, 1.571, 2.356, −0.524, 2.618) radians. Path planning experiments were conducted within this environment, involving the comparison of three distinct algorithms.

**Figure 12:**
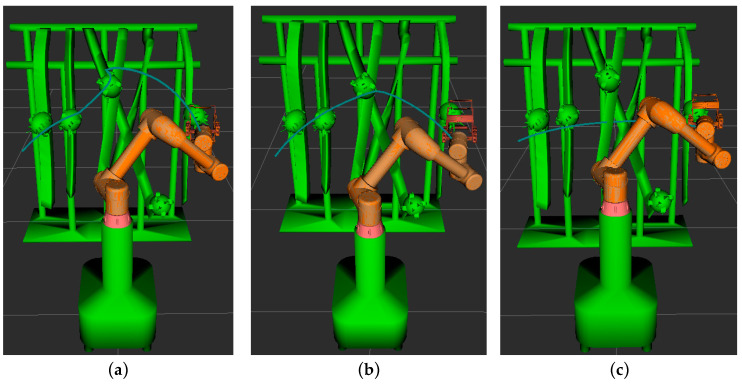
Simulated path trajectories for each algorithm. (**a**) RRT* algorithm; (**b**) APF-RRT* algorithm; (**c**) XN-RRT* algorithm.

**Figure 13:**
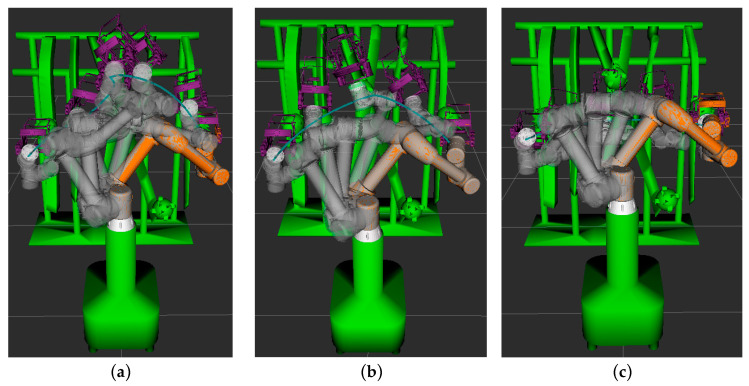
Simulation path planning results of each algorithm. (**a**) RRT* algorithm; (**b**) APF-RRT* algorithm; (**c**) XN-RRT* algorithm.

The process of path planning for the picking robot under various algorithms is illustrated in Figure 12. In the figure, the blue curve denotes the trajectory of the robot’s picking end-effector. The motion path of the robot for each algorithm is depicted by continuous white models, as shown in Figure 13. To evaluate the performance of each algorithm in the robot’s path planning, 100 independent tests were conducted for each algorithm within the simulation environment, and the means of the performance metrics were computed, as shown in Table 3.

As illustrated in Figure 12 and Figure 13, it is evident that, during the process of the robotic arm traversing from the leftmost pitaya to the target pitaya on the right, the path planned by the enhanced RRT* algorithm not only successfully avoids obstacles but also accomplishes the fruit-picking task. This path exhibits a notably smoother trajectory compared to those generated by the other two algorithms. As presented in Table 3, the XN-RRT* algorithm achieves the shortest path length of 978.91 mm, accompanied by a computation time of 0.524 s and a path planning success rate of 98%.

**Figure 14:**
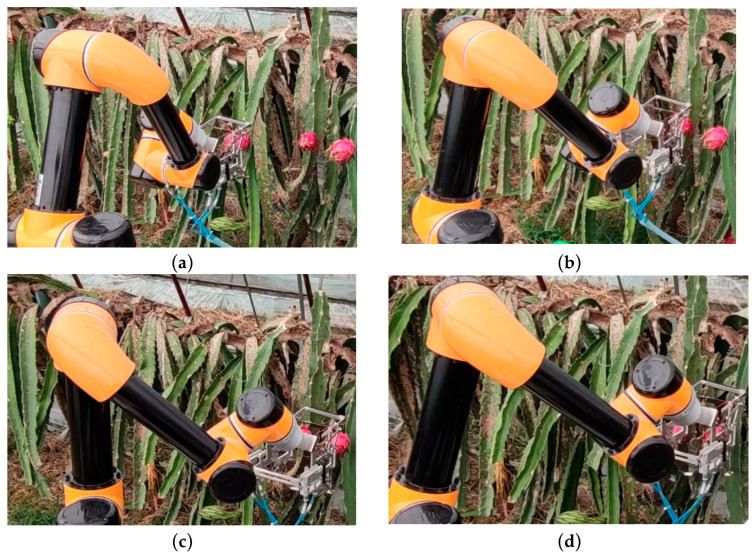
Actual obstacle avoidance path for dragon fruit picking. (**a**) Initial posture; (**b**) obstacle avoidance posture 1; (**c**) obstacle avoidance posture 2; (**d**) reached target attitude.

To validate the practical applicability of the algorithm presented in this study for real-world scenarios, path planning verification experiments were conducted on a robotic picking arm within a pitaya plantation environment, yielding the obstacle avoidance movement trajectory of the robotic arm, as shown in Figure 14. The experiment confirmed that, in real-world applications, the algorithm enables the robotic picking arm to effectively avoid obstacles, such as branches and fruits, and to navigate from the initial fruit to the target fruit, thereby successfully completing the obstacle avoidance path planning task.

## 6. Conclusions

(1) In addressing the path planning problem for dragon-fruit-picking robotic arms operating in complex environments, the XN-RRT* algorithm is introduced. This approach incorporates a normal-distribution-based sampling strategy and an enhanced artificial potential field (APF) guidance technique to facilitate node generation. Additionally, a greedy algorithm is employed to eliminate redundant nodes, and cubic B-spline curves are applied to optimize the path. These methods effectively mitigate the randomness of sampling points, reduce the overall path length, and enhance path smoothness.
(2) The results of the simulation tests demonstrate that the XN-RRT* algorithm outperforms both the traditional RRT* and APF-RRT* algorithms in terms of path quality and smoothness. Notably, it excels in search time, path length, node utilization efficiency, and path stability, thereby showcasing its superior performance in complex spatial path planning.
(3) The results from the dragon fruit picking experiment indicate that the XN-RRT* algorithm achieves a path planning success rate of 98%. Compared to the RRT* algorithm, the running time is reduced by 90.32%, the path length is shortened by 27.12%, and the planning success rate is improved by 14%. These findings substantiate the effectiveness and practical applicability of the XN-RRT* algorithm.

## Figures and Tables

**Table 1:** Test path planning data statistics of three algorithms in different environments.

Environment	Algorithm	Average Path Length/m	Average Running Time/s	Average Iteration Times
Simple discrete environment	RRT* algorithm	12.064	4.401	465.3
	APF-RRT* algorithm	11.821	1.387	224.7
	XN-RRT* algorithm	11.152	0.723	89.2
Complex narrow environment	RRT* algorithm	12.148	5.582	561.5
	APF-RRT* algorithm	12.115	1.885	262.3
	XN-RRT* algorithm	11.741	0.842	118.4

**Table 2:** Test path planning data statistics of three algorithms in 3D environment.

Environment	Algorithm	Average Path Length/m	Average Running Time/s	Average Iteration Times
3D environment	RRT* algorithm	2506.068	29.548	1698
	APF-RRT* algorithm	2363.286	9.764	846
	XN-RRT* algorithm	2215.865	1.118	114

**Table 3:** Picking test results.

Algorithm	Path Length/mm	Running Time/s	Success Rate/%
RRT* algorithm	1343.27	5.412	84
APF-RRT* algorithm	1251.64	2.746	90
XN-RRT* algorithm	978.91	0.524	98

## Data Availability

The data in this study are available upon request from the corresponding author.
